# Supplementary material for: Encouraging improvement in HPV vaccination coverage among adolescent girls in Kampala, Uganda
Source: PLoS One. 2022 Jun 9;17(6):e0269655. doi: 10.1371/journal.pone.0269655 (PMC9182299; doi:10.1371/journal.pone.0269655)
Supplement: S2 Appendix — (DOCX) [file pone.0269655.s002.docx]

**Appendix 2: Clinical characteristics of study participants**

| **Characteristics** | **Frequency** | **Percentage** |
| --- | --- | --- |
| **Weight in Kg**  Median (IQR) 42.7 (36.2, 52.8) | | |
| **Height in Cm**  Mean (SD) 150.6 (9.7) | | |
| **BMI**  Median (IQR) 18.7 (16.9, 21.7) | | |
| **Liking the body** |  |  |
| Yes | 193 | 67.0 |
| No | 8 | 2.8 |
| Missing | 87 | 30.2 |
| **Mood status** |  |  |
| Very happy | 54 | 18.8 |
| Happy | 110 | 38.2 |
| Neutral | 26 | 9.0 |
| Sad | 5 | 1.7 |
| Missing | 93 | 32.3 |
| **Chronic disease** |  |  |
| Yes* | 19 | 6.6 |
| No | 175 | 60.8 |
| Missing | 94 | 32.6 |
| **Reason for clinic visit** |  |  |
| Vaccination | 164 | 56.9 |
| Health Education | 12 | 4.2 |
| Sick | 6 | 2.1 |
| Medical check-up | 6 | 2.1 |
| Vaccination & Health Education | 12 | 4.2 |
| Sick & Vaccination | 4 | 1.4 |
| Missing | 84 | 29.2 |
| **Ever had sex** |  |  |
| Yes | 4 | 1.4 |
| No | 186 | 64.6 |
| Missing | 98 | 34.0 |
| **HIV testing** |  |  |
| Yes | 27 | 9.4 |
| No | 166 | 57.6 |
| Missing | 95 | 33.0 |
| **HIV status** |  |  |
| Infected | 1 | 3.7 |
| Uninfected | 21 | 77.8 |
| Unknown | 5 | 18.5 |

**Epilepsy, asthma, allergic conjunctivitis, dysmenorrhea, cancer, ulcers, STI, UTI, unspecified allergies*
