# Supplementary material for: Encouraging improvement in HPV vaccination coverage among adolescent girls in Kampala, Uganda
Source: PLoS One. 2022 Jun 9;17(6):e0269655. doi: 10.1371/journal.pone.0269655 (PMC9182299; doi:10.1371/journal.pone.0269655)
Supplement: S3 Appendix — (DOCX) [file pone.0269655.s003.docx]

**Appendix 3 – Data Collection Tools for Girls and Caretakers**

**DATA ABSTRACTION FORM – for retrospective quantitative data for girls aged 9-14 years for the period November 2015 to November 2017**

Study No: …………… Date of Evaluation: …..../…../………..

Day/Month/Year

Date enrolled in clinic ….../…../……….

**Socio-Demographic characteristics**

Name: …………………………………………………………

Age: ……………. Date of Birth: ……………………………..

Height: ………………. Weight: ……………………

Address: village ……………………….. District ……………………………

Currently in school a) Yes b) No

Class: …………….

Guardian a) Both mother and father

b) Mother alone

c) Father alone

d) Grandparents

e) Other ………………………….

Date of first dose received: ……………………………..

Was the second dose received? …………………………

Date second dose received: **……………………………..**

Tribe ………………………………………….

Religion ………………………………………

Reason of clinic visitation ………………………………………………………..

Presence of chronic disease ……………………………………………………………

HIV test: 1) Yes

2) No

HIV status: Infected ………….

Uninfected ……….

Unknown …………

Do you like your body 1) Yes

2) No

School performance: 1) very good

2) Good

3) Average

4) Poor

Future goals ………………………………………………………………..

Have you ever had sex: 1) Yes

2) No

Current mood: 1) Very happy

2) Happy

3) Neutral

4) Sad

**Appendix 8**

**ABSTRACTION FOR CARETAKERS**

Study No: …………… Date of Evaluation: …..../…../………..

Day/Month/Year

Date enrolled in clinic ….../…../……….

**Socio-Demographic characteristics**

Name: …………………………………………………………

Age: ……………. Date of Birth: ……………………………..

Level of education: ……………………………………………..

Occupation: ……………………………………………………..

Marital status: …………………………………………………..

Address: village ……………………….. District ……………………………

No. of children: ………………………………………………….
