# Supplementary material for: Encouraging improvement in HPV vaccination coverage among adolescent girls in Kampala, Uganda
Source: PLoS One. 2022 Jun 9;17(6):e0269655. doi: 10.1371/journal.pone.0269655 (PMC9182299; doi:10.1371/journal.pone.0269655)
Supplement: S4 Appendix — (DOCX) [file pone.0269655.s004.docx]

**Appendix 4 – Additional data collection (and FGD) tools**

Good morning/ afternoon,

Thank you all for agreeing to participate in this study.

My name is …………………………….. and this is my colleague …………………………. This study is being carried out by Dr Patrick Lydia, a postgraduate student in the Department of Pediatrics and Child Health, Makerere University.

We are conducting several discussions to determine the factors associated with HPV vaccine completion among girls9-14 years. Your opinions are very important; please feel free to share your concerns and ideas about the topic that comes up in today’s discussion. Your responses will serve as basis to improve HPV vaccine completion levels in the country.

The discussion will take between 30 – 45 minutes. All information you provide will be confidential. There are no wrong answers and you do not have to agree with what others say during the discussion. We ask for permission to audio record this session because we do not want to miss any of your comments. There will be no names attached to your views.

Attain general information: (for adolescents these will be attained from the files, information from caretakers will be obtained prior to the interview)

1. Have you ever heard about HPV?
2. What have you heard about HPV? *(probe for HPV transmission, what HPV causes, HPV prevention if not mentioned)*
3. Why is vaccination against HPV done? (*Probe for importance of vaccine if not mentioned.)*

*Have you heard of HPV vaccine, if yes what are your sources and what have you heard about it?*

1. Are there any adolescents you know who have not initiated or completed the HPV vaccination? What are some of the reasons?

*Probe for barriers to HPV vaccination – age of adolescent, parental consent, fear of side effects, inadequate information about vaccine, sexual activity/risky sexual behaviour, HCW recommendation, availability of vaccine, boarding vs day school*

1. What would you say encouraged you to receive the HPV vaccine?

*Probe on the motivators of HPV vaccination – HCW recommendation, gender of HCW, awareness of HPV vaccine benefits, parental wishes or peer influence, sexual activity, perceived risk of HPV infection*

1. What do you think should be done to improve uptake and completion of HPV vaccine in Uganda?
2. Do you have any other comment or questions you would like us to talk about in relation to HPV vaccine?
