# Supplementary material for: Encouraging improvement in HPV vaccination coverage among adolescent girls in Kampala, Uganda
Source: PLoS One. 2022 Jun 9;17(6):e0269655. doi: 10.1371/journal.pone.0269655 (PMC9182299; doi:10.1371/journal.pone.0269655)
Supplement: Attachment — Submitted filename: Response to Reviewers.docx [file pone.0269655.s005.docx]

15^th^ September 2021

**Re: Response to Reviewers -** manuscript entitled **‘Encouraging improvement in HPV vaccination coverage among adolescent girls in Kampala, Uganda**’ We have responded to the comments of reviewers as follows:

**Reviewer #1:**

1. Introduction would benefit from updated data on US HPV vaccine uptake. the citations presented are old.

*Done, included over 10 new updated references*
2. In the results, you mention that the age of adolescents ranged 9-14, but that was the inclusion criteria, so it seems odd to present it this way. It is possible that ages may not have been included, but that would be a more appropriate way to report - e.g. "All ages in the eligibility criteria (9-14) were represented by adolescents in the study"

*Done, recommended change included*
3. The finding about mood is confusing - what is the model by which this very acute measure at the time of the study could impact vaccination status? Additionally, for the final items included, can you comment more on the clinical significance of including them?

*Done, explanation and reorganization included*
4. Were all of the themes similar between parents and adolescents? Or were some more common in one group than another?

*Discussed in text, and improved*
5. The discussion is mostly a list of other papers, but does not have a clear synthesis of all of the prior data and your findings. it's presented more like a review than like a clear discussion.

*Done, discussion fully redone with new updated citations*
6. Were there any a priori sample size estimates generated, since N was identified as a limitation?

*Sample calculations done, but decision made to review all folders available this being a retrospective study and not many patients in the clinic.*

**Reviewer #2:**

The paper “Encouraging improvement in HPV vaccination coverage among adolescent girls in Kampala, Uganda” addressed the topic of completion of the full cycle of vaccination against HPV. This is an interesting topic and the evaluation of underlying reasons of completion/uncompletion is important. Nonetheless, the papers show some inconsistencies and problems that are reported hereafter.

Abstract
1. The abstract would need including more information in order to make the approach and, therefore, the conclusion clearer.

*Done, included*
Introduction
2. Introduction needs to be deeply rephrased in order to avoid redundancy (need for two doses) and provided with more reference and data to support the need to assess vaccine completion and understand its determinants. The rationale of the study is not well framed. Furthermore, some parts are vague or miss time reference (i.e., “Two strategies were proposed, and some training was conducted for some health care workers. Based on anecdotal report by Global Alliance for Vaccines and Immunization full country evaluations (GAVI FCE) report 2016 poor national vaccine uptake and completion has been noted [5]. Uptake of HPV-1 in Uganda is at 73.8% while that of the second dose (HPV-2) at 17.3%”).

*Introduction has been extensively redone, new references included, and the recommended changes added*
Methods
3. Authors quoted “The target population of the study was all girls aged 9-14 years in Kampala, Uganda and included all girls aged 9-14 years attending the adolescent clinic and girls who received the first dose at-least in the last 6 months in the clinic”: the time reference is not clear as the Authors further reported that “The principal investigator screened all files in the adolescent clinic from November 2015 to July 2017”.

*Done, these corrections have been added in the extensive review of the paper to reflect reviewer recommendations*
4. Another point that needs to be addressed is the following: “Those who received the second dose 5-6 months after the 1st dose was then selected. “This is not true because the study population was represented by girls receiving the first dose. The vaccine schedule completion within 5-6 months was the primary endpoint of the study.

*Done, this error has been corrected in text*
5. Authors quoted “Incidence risk ratios for the factors were calculated with their 95% confidence intervals.”. Which variables did the Authors consider for this analysis? Statistical methods for the bivariate and multivariable analysis should be described.

*Done, this error has been corrected in text*
6. The description of qualitative part should be made more fluent, avoiding redundant information (number of focus group performed, composition of groups, etc).
*Done, most repetitive text has been redacted*
Results
7. Please provide the readers with a caption explaining the variable “class” and illustrate how school performance and mood were evaluated first and then extracted.

*Done, explanation provided and this aspect discussed in the discussion section*
8. Table 3 should be made more readable as the name of variables are undistinguishable from categories and each variable should be provided with the number of observations with available information.

*Done, this has been corrected, table moved to supplementary documents*
9. The results on the relationship between schooling status and HPV-2 completion were not significant. Please revise the text accordingly.

*Done, this error has been corrected in text*
10. Results also includes some methodological aspects (variables entered in the multivariable analysis, evaluation of confounding). All these elements should be moved in methods. Furthermore, please keep in mind that because of the nature of the study no causal inference can be done. Indeed, why did the authors pay attention to confounding?

*Done, this error has been corrected in text*
11. Motivations discussed under “Health Seeking Behaviors” seem to fall in peer influence.

*Done, this error has been corrected in text*
12. In the reporting of qualitative data Authors quoted “Quantitative data indicates that majority of the adolescents (63%) attended the clinic for vaccination”. What does it refer to?

*Done, this error has been corrected in text*
Discussion
13. Authors quoted “National completion levels for 2016, HPV-1 and HPV-2 was at 83% and 22% respectively while in 2017 HPV-1 was at 72% and 28% [5, 11]. The difference could be explained by the different models used in delivery of the vaccine i.e., school-based and age-based strategies, lack of follow-up for the second doses, inadequate information and guidance on eligibility criteria”. Please, provide more information about the calculation of these national coverage data.

*Done, the discussion has been extensively redone, corrections have been made, references updated to reflect the comments and guidance of the reviewers*
14. There is an extensive discussion on the results coming from the qualitative analysis, but I am concerned about the fact that there was not any attempt to quantify themes and sub-themes recurrence among different groups. This is a major problem that prevents making final conclusions on the association between specific factors and completion. I would invite Authors to address this problem.

*Efforts have been made to address this matter through the extensive revision of the discussion to reflect the comments and guidance of the reviewers. We are ready to modify this more to the satisfaction of the reviewers*
15. The study population in my opinion might be affected by selection bias and generalizability of results should be discussed further.

*This limitation has been acknowledged, especially bearing in mind the retrospective nature of the study*
