# Supplementary material for: Encouraging improvement in HPV vaccination coverage among adolescent girls in Kampala, Uganda
Source: PLoS One. 2022 Jun 9;17(6):e0269655. doi: 10.1371/journal.pone.0269655 (PMC9182299; doi:10.1371/journal.pone.0269655)
Supplement: Attachment — Submitted filename: PLOS Review.docx [file pone.0269655.s006.docx]

**PONE-D-21-09701
"Encouraging improvement in HPV vaccination coverage among adolescent girls in Kampala, Uganda"**

This was analyses of factors related to low HPV vaccination in Uganda. The authors reported on factors associated with completion of the vaccine series. This paper may be of interest to researchers interested in childhood vaccinations and how to promote higher HPV vaccination rates.

Comments

Abstract

Methods – Change data was to “data were”

Conclusion – what are the implications of your findings? What strategies or interventions could help achieve higher rates?

Introduction, p. 3

2^nd^ paragraph,

- Not certain why US rates are presented. If the authors want to draw parallels to Uganda then recent rates of vaccinations for girls should be presented there as well
- word missing from statement: *There has been poor HPV vaccine uptake since its introduction into the national immunization program of .*

last paragraph

- what strategies or interventions did your ministry employ? Do they correspond with WHO guidelines or Community Guide to Preventive Services to increase vaccination rates?

p. 4, first paragraph

- needs a period at end of first sentence

Methods

- What type of mixed methods design was this study? (concurrent, sequential). Please describe it in the methods.
- Please describe if the vaccines are free of charge. This may be implicit but please make it explicit in this statement, if this is true. *All vaccinations are supplied through the Ministry of Health’s EPI program*

P. 5, study procedures

- Change sentence to say “data… were”; need to change that for the rest of the paper as well
- What was the multivariate analysis? Need to say the tests in methods
- Last paragraph. Last sentence is a run on sentence; add “and” or “;” or make 2 sentences

p. 6, first paragraph

- would recommend describing analyses and citing a reference to match your analyses (deductive based on interview guide, thematic?
- Indicate initials from co author lists of the coders of the transcript or who did the analyses

P. 7, ethical consideration

Last sentence, this is not needed: *The findings are presented in this manuscript and in the supporting information files provided*

Results, p. 8

- First paragraph, first sentence is a run on sentence
- Need to define what you are considering early vs late completion. Put in into the methods or in parentheses here
- You may want to add other characteristics of the included girls into this paragraph

Table 2

Suggest putting the N into the Title

p. 10,

first sentence, would move Table 3 to the end of the sentence. *The factors associated with timely completion of HPV-2 at bi-variable analysis were (Table 3)*

Table 3

Would put the key variables in bold like you did for Table 2

Table 5, page 13

Could you indicate if the key themes arose from the FGD with the girls, parents or both. That would give a better sense of the strength of the themes

p. 16,

change *One went ahead* and said: to “One commented”

p. 19

- could remove 2^nd^ sentence and put that under methods for what was consider timely: Completion at 6 months was considered timely
- what were your goal for vaccination of girls in Uganda or by WHO; could present that in the sentence: *this figure still fell below the recommended national average for countries by WHO*
- you discuss that facilities do not have strategies for reminders; what are the implications for this in terms of practice?
- Since your data may be difficult to compare to other countries, could you speak to other vaccinations for children and how this vaccination rate is and what parallels could be drawn?
- In the Discussion, could you describe any future research you would seek after the conduct of this study?

Conclusion

- First sentence; data do not need the parentheses
- Perhaps add a sentence for what practice implications would increase the compliance with vaccine completion
