# Supplementary material for: Encouraging improvement in HPV vaccination coverage among adolescent girls in Kampala, Uganda
Source: PLoS One. 2022 Jun 9;17(6):e0269655. doi: 10.1371/journal.pone.0269655 (PMC9182299; doi:10.1371/journal.pone.0269655)
Supplement: Attachment — Submitted filename: Response to Reviewers.docx [file pone.0269655.s007.docx]

10^th^ March 2022

**Re: Response to Reviewers -** manuscript entitled **‘Encouraging improvement in HPV vaccination coverage among adolescent girls in Kampala, Uganda**’ We have responded to the comments of reviewers as follows:

**Abstract**

Methods – Change data was to “data were” **- DONE**

Conclusion – what are the implications of your findings? What strategies or interventions could help achieve higher rates? - DONE

Introduction, p. 3

2^nd^ paragraph,

- Not certain why US rates are presented. If the authors want to draw parallels to Uganda then recent rates of vaccinations for girls should be presented there as well – CORRECTED
- word missing from statement: *There has been poor HPV vaccine uptake since its introduction into the national immunization program of .* – CORRECTED

last paragraph

- what strategies or interventions did your ministry employ? Do they correspond with WHO guidelines or Community Guide to Preventive Services to increase vaccination rates? QUESTION HAS BEEN RESPONED TO IN TEXT.

p. 4, first paragraph

- needs a period at end of first sentence – CORRECTED

Methods

- What type of mixed methods design was this study? (concurrent, sequential). Please describe it in the methods. DONE
- Please describe if the vaccines are free of charge. This may be implicit but please make it explicit in this statement, if this is true. *All vaccinations are supplied through the Ministry of Health’s EPI program DONE*

P. 5, study procedures

- Change sentence to say “data… were”; need to change that for the rest of the paper as well DONE
- What was the multivariate analysis? Need to say the tests in methods. CORRECTED
- Last paragraph. Last sentence is a run on sentence; add “and” or “;” or make 2 sentences DONE

p. 6, first paragraph

- would recommend describing analyses and citing a reference to match your analyses (deductive based on interview guide, thematic? DONE
- Indicate initials from co author lists of the coders of the transcript or who did the analyses DONE

P. 7, ethical consideration

Last sentence, this is not needed: *The findings are presented in this manuscript and in the supporting information files provided RETAINED, BECAUSE IT IS AN EDITORIAL REQUIREMENT FOR PLOS ONE*

Results, p. 8

- First paragraph, first sentence is a run on sentence NOTED, CORRECTED
- Need to define what you are considering early vs late completion. Put in into the methods or in parentheses here DONE
- You may want to add other characteristics of the included girls into this paragraph DONE

Table 2

Suggest putting the N into the Title DONE

p. 10,

first sentence, would move Table 3 to the end of the sentence. *The factors associated with timely completion of HPV-2 at bi-variable analysis were (Table 3)* DONE

TABLE 3

Would put the key variables in bold like you did for Table 2 DONE

TABLE 5, PAGE 13

Could you indicate if the key themes arose from the FGD with the girls, parents or both. That would give a better sense of the strength of the themes DONE

p. 16,

change *One went ahead* and said: to “One commented” CORRECTED

p. 19

- could remove 2^nd^ sentence and put that under methods for what was consider timely: Completion at 6 months was considered timely. DONE
- what were your goal for vaccination of girls in Uganda or by WHO; could present that in the sentence: *this figure still fell below the recommended national average for countries by WHO. DONE*
- you discuss that facilities do not have strategies for reminders; what are the implications for this in terms of practice? THIS HAS BEEN EXPLAINED FURTHER
- Since your data may be difficult to compare to other countries, could you speak to other vaccinations for children and how this vaccination rate is and what parallels could be drawn? THIS HAS BEEN EXPLAINED FURTHER
- In the Discussion, could you describe any future research you would seek after the conduct of this study? THIS HAS BEEN EXPLAINED FURTHER

Conclusion

- First sentence; data do not need the parentheses CORRECTED
- Perhaps add a sentence for what practice implications would increase the compliance with vaccine completion DONE
